# A Review of Medicinal Plants Used for Uterine Disorders in Makhzan al-Adwiyyah: Insights from Persian Medicine

**DOI:** 10.5812/ijpr-160193

**Published:** 2025-09-07

**Authors:** Fatemeh Rabizadeh

**Affiliations:** 1 Department of Biology, Farzanegan Campus, Semnan University, Semnan, Iran

**Keywords:** Traditional Medicine, Uterus, Medicinal Family, Temperament, Plants

## Abstract

**Context:**

Uterine disorders are among the most prevalent gynecological problems in women. Persian Medicine (PM) has long provided herbal approaches for managing such conditions, based on temperament theory and humoral balance.

**Objectives:**

This study aimed to identify medicinal plants used in PM for the treatment of uterine disorders and to assess their pharmacological evidence.

**Methods:**

A literature review of classical PM texts, including Makhzan al-Adwiyyah and Taqwim al-Abdan, was conducted to extract herbs recommended for uterine ailments. Plant names, traditional indications, and temperament properties were documented. Modern pharmacological evidence for each plant was then retrieved from scientific databases (PubMed, Scopus, Web of Science).

**Results:**

A total of 97 plant species belonging to 39 botanical families were identified. Most herbs exhibited hot and dry temperaments, aligning with the PM view that uterine disorders are linked to cold-moist imbalances. Common traditional indications included dysmenorrhea, endometrial inflammation, discharge, and cold uterus. In this study, the pharmacological findings for 10 key species, including *Nigella sativa*, *Crocus sativus*, and *Vitex agnus-castus*, were summarized.

**Conclusions:**

Several PM herbs show promising potential for treating uterine disorders and align with modern pharmacological mechanisms. These findings provide a basis for further research and may support the development of evidence-based herbal therapies for women’s reproductive health. Among the most frequently cited species, *Foeniculum vulgare*, *V. agnus-castus*, and *N. sativa* exhibit promising pharmacological activity, have shown potential uterotonic effects, and may support menstrual regulation, relieve uterine pain, and help modulate hormonal balance. The findings of this review may support the design of novel plant-based interventions in reproductive medicine, guided by both traditional knowledge and modern scientific evidence.

## 1. Context

Medicinal plants have long been used in various cultures for the treatment of human diseases. Today, herbal therapies continue to play an important role in complementary and integrative medicine. A significant number of these remedies are directed toward managing gynecological conditions, including menstrual irregularities, uterine pain, and reproductive disorders. Interest in natural approaches to women's health is increasing, especially in response to concerns about the side effects and long-term risks of synthetic drugs (1-4).

Persian medicine (PM), with a history spanning over a thousand years, played a major role in shaping medical practices from the Middle Ages to the Renaissance and served as a model for medical schools during the Islamic Golden Age (5). Classical texts such as Avicenna's Canon of Medicine and Al-Razi's Al-Hawi provide detailed accounts of treatments for women’s diseases (6-8). The PM emphasizes health and disease through the theory of mizaj (temperament), rooted in the balance of four qualities: Hot, cold, wet, and dry. Each organ, including the uterus, is believed to possess a specific temperament, and disturbances in this balance are thought to cause disease. For example, a "cold" uterus may be associated with delayed menstruation or infertility, while a "hot" uterus might present with excessive bleeding or inflammation. Treatment aims to restore the natural temperament through herbs, diet, and lifestyle adjustments (9).

In PM, uterine diseases are treated promptly to prevent recurrence and systemic involvement, particularly liver weakness. The uterus is considered closely connected to other organs, especially the brain. According to classical Persian sources, there are 41 recognized uterine disorders and four major types of uterine temperament imbalances: Hot, cold, cooler, and dry (9). Similar temperament-based medical systems, such as Traditional Chinese Medicine (TCM), also adopt a holistic approach to women’s health.

Zhou et al. demonstrated that TCM is effective in managing gynecological and obstetric conditions such as menstrual irregularities, infertility, and postpartum complications. Common TCM modalities include acupuncture, herbal medicine, and dietary therapy, which aim to restore systemic balance and improve quality of life (10, 11). The TCM techniques like acupuncture stimulate energy flow, while herbal remedies and dietary therapies promote physiological healing and resilience.

In PM, conditions such as "hypoestrogenism", "endometrial inflammation", and "leukorrhea" are often treated with herbs categorized as hot and dry. These traditional terms can conceptually align with modern gynecological disorders like hypoestrogenism, endometrial inflammation, fibroids, leukorrhea, or cervicitis. Such interpretations facilitate interdisciplinary understanding and bridge traditional with biomedical frameworks. For example, plant extracts like *Radix rehmanniae* and *Fructus lycii* have shown potential in restoring ovarian function in infertile patients with fewer side effects compared to synthetic drugs (10). In PM, temperament-based classifications are employed to explain physiological and pathological states of the body. Temperament (mizaj) refers to a set of qualitative characteristics that influence health and disease. These temperaments — hot, cold, dry, and moist — are traditionally defined based on observable traits. A hot temperament implies increased warmth, activity, and circulation, which can be likened in modern biomedical terms to elevated metabolic rate or inflammatory responses. In contrast, a cold temperament reflects sluggishness and a lack of internal warmth, analogous to hormonal suppression or poor circulation. A dry temperament is associated with roughness, stiffness, and a lack of internal moisture, corresponding to dehydration, tissue rigidity, or catabolic conditions. Lastly, a moist temperament signifies an excess of bodily fluids, softness, and looseness — features that resemble modern clinical signs such as edema, fluid retention, or mucous accumulation. These parallels illustrate how traditional medical concepts can be interpreted within contemporary biomedical frameworks. Furthermore, traditional approaches may help mitigate the side effects of surgical interventions such as hysterectomy and enhance quality of life.

Globally, approximately 80% of the population relies on traditional herbal medicine, and many modern drugs have originated from plant-based sources. These therapies remain popular in countries like India and China due to their accessibility, affordability, and relative safety (12, 13). Indian traditional medicine, for instance, employs herbs like *Moringa oleifera* and *Azadirachta indica* for gynecological care (14, 15).

In Iran, integrating traditional knowledge with modern medicine offers an opportunity to scientifically validate the country’s rich medicinal flora. The aim of this study is to identify medicinal plants used in PM for treating uterine and women’s health disorders and to compare them with those used in Chinese and Indian systems, thereby contributing to safer and more effective complementary therapies.

## 2. Methods

This review was conducted by systematically searching classical PM texts, including Makhzan al-Adviyeh by Aghili Khorasani (16) and Taghvim al-Abdan fi Tadbir al-Ensan by Ibn Jazlah (17). Although other sources like Kamil al-Sanaʿah and Zakhirah-i Khwarazmshahi were reviewed, they lacked clear herb-to-disease mappings. Makhzan al-Adviyeh was prioritized for its structured format and explicit references to uterine effects. Medicinal plants were selected based on direct relevance to uterine conditions and reliable identification of their scientific names through authoritative botanical sources (18, 19).

The PM often uses temperament-based terminology to describe gynecological conditions. For biomedical clarity, traditional terms are mapped to approximate modern equivalents: "Hypoestrogenism" may indicate hypoestrogenism or uterine atony; "endometrial inflammation" may refer to fibroids or inflammation; "leukorrhea" may correspond to abnormal secretions. These mappings are used throughout this manuscript to enhance clinical and scientific accessibility.

### 2.1. Inclusion and Exclusion Criteria

Plants were selected based on the following criteria: (1) Direct mention in PM sources for uterine or gynecological disorders; (2) confirmed botanical identification at the species level. Priority was given to those with documented pharmacological actions (e.g., emmenagogue, anti-inflammatory, uterine tonic) and at least one modern pharmacological or clinical study. Plants were also assessed based on the credibility and quality of the sources, ensuring that the documentation was reliable and scientifically valid.

Plants were excluded if they (1) lacked sufficient documentation on their uterine application, (2) had uncertain botanical identity, or (3) were considered highly toxic, obsolete, or banned in modern pharmacopoeias. Highly toxic species like *Aristolochia* spp. were discussed only in the safety section and excluded from efficacy evaluations. Additionally, plants without adequate modern pharmacological or clinical evidence were not included, even if they were mentioned in classical texts, unless new clinical or experimental data validated their relevance.

Evidence levels were categorized as Strong, moderate, or weak based on three main criteria: (1) The number of published studies, (2) the type of evidence (human clinical data given the highest weight), and (3) reproducibility and consistency across studies.

**Figure 1.**
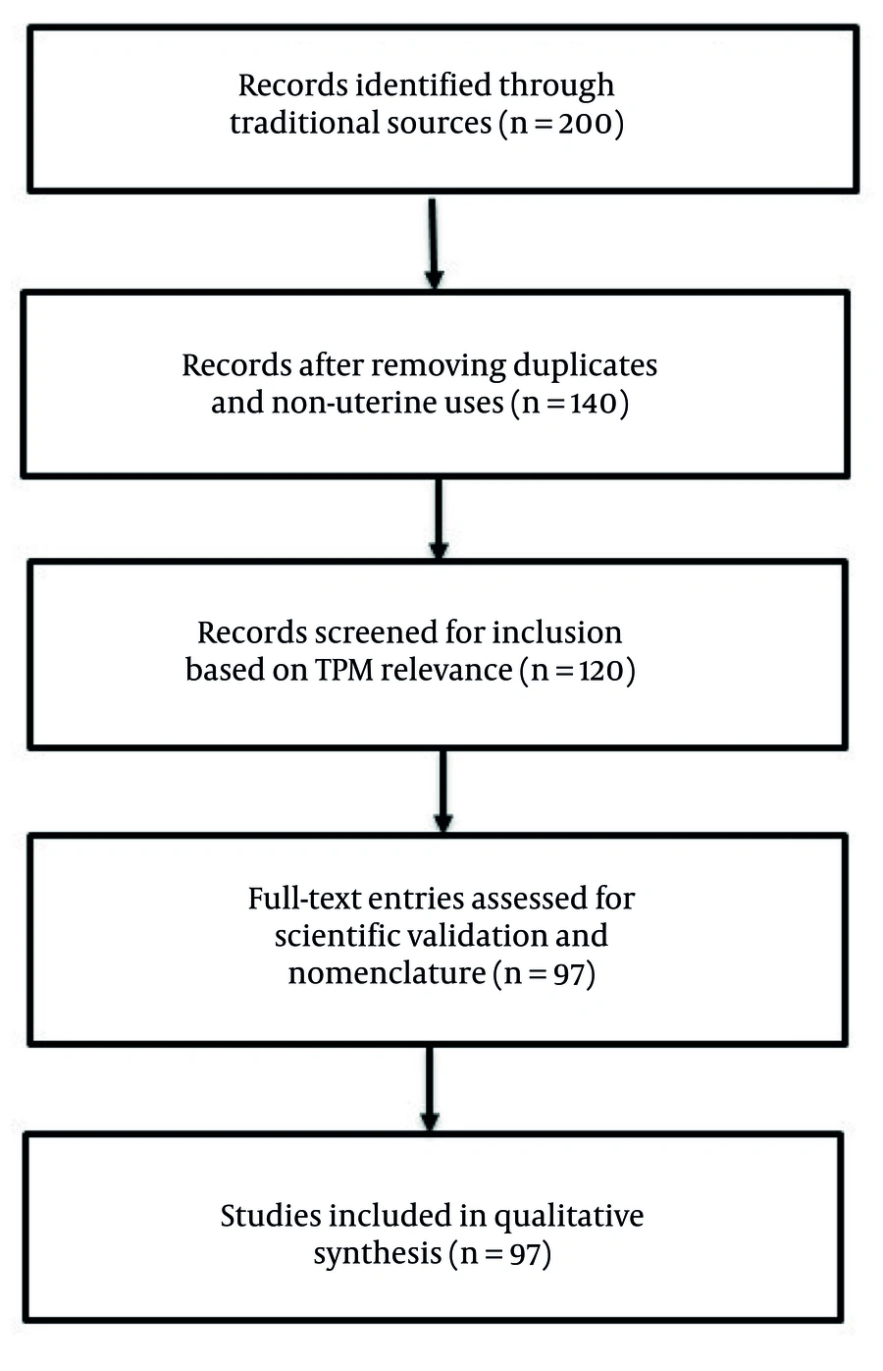
PRISMA-style flow diagram illustrating the selection process of medicinal plants used in Persian medicine (PM) for the treatment of uterine disorders. From an initial pool of 200 plants identified in Makhzan al-Adviyeh and Taqvim al-Abdan, a final set of 97 plants was selected based on traditional indications, relevance to uterine conditions, and validated scientific nomenclature.

To ensure scientific validity and transparency, a structured workflow was followed, and a PRISMA-style flow diagram (Figure 1) was used to document the identification, screening, and inclusion process. Temperament classifications were based on explicit descriptions in PM texts and categorized into four major types: Hot, cold, dry, and moist, as well as their combinations. The temperament types were categorized using the specific language and principles described in classical sources, with careful attention to how these temperaments influence the therapeutic properties of the plants.

In the second phase, complementary pharmacological data for the selected plants were retrieved from databases including PubMed, Scopus, and Web of Science using Boolean combinations of keywords such as: "Uterine" OR "gynecological" OR "menstrual" OR "female reproductive" AND ("plant name" OR "botanical name") AND ("activity" OR "effect"). Both scientific names and common synonyms were searched.

- Inclusion criteria were: (1) Studies on female reproductive health; (2) in vivo, in vitro, or clinical studies using identified species; (3) English-language publications from 2000 - 2024.

- Exclusion criteria were: (1) Reviews without original data; (2) studies lacking dosage, species confirmation, or gynecological relevance.

On average, 3 - 5 articles per plant were reviewed. Table 1 reflects the evidence ranking (strong, moderate, weak) based on these studies.

**Table 1.** List of Medicinal Plants Used in Traditional Medicine to Treat Uterine Diseases

Scientific Name	Bioactive Compound(s)	Type of Study	Uterine-related Efficacy	Primary Uterine Use (Yes/No)	Level of Evidence	Ref.
***Berberis vulgaris***	Berberine, palmatine	In vivo, in vitro	Anti-inflammatory and antimicrobial effects beneficial for abnormal discharge	No	Moderate	(20)
***Artemisia absinthium***	Artemisinin, thujone	In vivo	Antispasmodic and anti-inflammatory in uterine pain models	Yes	Moderate	(21)
***Ruta graveolens***	Rutin, quercetin, volatiles	In vivo	Emmenagogue and antispasmodic activity in uterus-related models	Yes	Weak	(22)
***Vitex agnus-** **castus***	Agnoside, flavonoids	Clinical	PMS and menstrual regulation; hormonal modulation in women	Yes	Strong	(23)
***Cannabis sativa***	THC, CBD	In vivo, Clinical	Analgesic and hormonal modulation; used in dysmenorrhea and pain management	No	Strong	(24)
***Cinnamomum zeylanicum***	Cinnamaldehyde, eugenol	In vitro, in vivo	Uterine stimulant; regulates menstruation via anti-inflammatory effects	No	Moderate	(25)
***Ajuga ** **chamaepitys***	Flavonoids, glycosides	In vitro	Suggested antimicrobial and anti-inflammatory for endometritis	Yes	Weak	(26)
***Teucrium ** **polium***	Flavonoids, diterpenes	In vivo	Uterine tonic; antioxidant and anti-inflammatory activity	Yes	Moderate	(27)
***Echium ** **amoenum***	Rosmarinic acid, flavonoids	In vivo	Anti-inflammatory and analgesic in pelvic pain models	No	Moderate	(28)
***Nigella sativa***	Thymoquinone	In vivo, Clinical	Anti-inflammatory and hormonal regulation in reproductive system	No	Strong	(29)

The strength of evidence was graded as follows: Strong – supported by clinical trials, animal models, and mechanistic studies; moderate – based on in vitro or animal studies without human trials; weak – based on limited data or traditional use without experimental confirmation. In cases where evidence was inconsistent or studies lacked methodological quality, the plant was conservatively placed in the "Weak" category.

This classification aimed to provide a clearer scientific context for the therapeutic potential of each plant and to assist researchers in identifying priority species for further investigation. The top 10 most frequently cited plants in PM were further analyzed for their bioactive compounds, in vitro/in vivo efficacy, and safety profiles based on published scientific studies (30). These databases were chosen for their comprehensive coverage of pharmacological and clinical research, ensuring the inclusion of the most reliable and up-to-date information.

This dual approach — classical textual analysis and modern literature screening — aims to build a bridge between traditional knowledge and contemporary evidence-based research. By integrating both sources of information, the study provides a more robust understanding of the medicinal plants' potential in treating uterine and gynecological disorders.

To ensure methodological rigor and transparency, a PRISMA-style workflow was employed to document the plant selection process. The steps were as follows:

#### 2.1.1. Identification

A total of 150 records were identified from classical PM sources, including Makhzan al-Adviyeh. These texts were chosen for their historical significance and their comprehensive references to uterine and gynecological conditions.

#### 2.1.2. Screening

After the initial review, 97 plants were selected based on their direct mention in relation to uterine disorders. The screening process ensured that only plants with clear references to gynecological applications were considered.

#### 2.1.3. Eligibility

Among these, 55 plants had available pharmacological or scientific literature supporting their bioactivity. These plants were subject to further analysis based on the availability of modern research that confirmed their therapeutic properties.

#### 2.1.4. Inclusion

Finally, 40 plants with confirmed pharmacological or clinical relevance were included in the detailed analysis. These plants were selected based on their documented effectiveness, with particular emphasis on those with clinical trials or significant pharmacological evidence supporting their use.

In traditional Persian medicine (TPM), uterine enemas (tanqiyah al-rahim) are considered therapeutic procedures used to relieve endometrial inflammation, discharge, or inflammation. Accordingly, when a plant is described as “useful for uterine enemas”, it refers to its role as an ingredient in medicinal preparations for such treatments, rather than being a treatment for an independent condition called “uterine enema”.

This study employed a structured narrative review approach in the field of ethnopharmacology. While some components of the PRISMA framework (such as the flowchart and structured database searches) were implemented to enhance transparency, the review is not a fully systematic review in the conventional sense. Instead, the emphasis was on integrating data from classical TPM texts with modern pharmacological literature to identify and analyze medicinal plants used in the treatment of uterine disorders. Although PRISMA-style methods were applied, the study is best categorized as a structured narrative review rather than a systematic review.

## 3. Results

Table 2 presents 97 medicinal plants traditionally used for treating uterine disorders, including their scientific names, traditional Persian names, temperaments, and key therapeutic properties. For example, *Artemisia absinthium* (absinthe), with a hot and dry temperament, is used for treating endometrial inflammation, while *Anthemis nobilis* (chamomile), also hot and dry, is known to alleviate uterine pain. The use of scientific nomenclature ensures accurate botanical identification and facilitates further pharmacological investigations. As shown in Table 2, the traditional disease terms are based on the theory and terminology of PM, which classifies uterine disorders according to imbalances in temperament (mizaj) — such as coldness, moisture, or excess bile/phlegm. Some of these conditions can be tentatively linked to modern biomedical diagnoses. Where possible, the table provides traditional terms alongside approximate clinical equivalents, although exact mapping is not always feasible. For instance, “uterine swelling” (endometrial hypertrophy/inflammation) may correspond to endometrial or cervical inflammation; “cold womb” (hormonal insufficiency/decreased uterine tone) may relate to hypoestrogenism or poor uterine circulation; and “excess uterine moisture” (vaginal or uterine discharge/secretory excess) may be comparable to leukorrhea or excessive cervical discharge. Similarly, “falling of the womb” can be roughly equated with uterine prolapse, while “uterine pain” could indicate dysmenorrhea or pelvic pain. The term “uterine gas” is symbolic of bloating or abdominal discomfort, but it does not represent a defined biomedical entity. These interpretations are provided to facilitate understanding for readers unfamiliar with PM and are not intended as exact diagnostic correspondences. Plants marked with the designated warning symbol in Table 2 are recognized as toxic or hazardous based on modern toxicological evidence; they are presented for historical reference only and should not be used clinically or experimentally without strict safety evaluation.

**Table 2.** Pharmacological Insights into Top Traditional Persian Medicinal Plants for Uterine Disorders: Bioactive, Efficacy, and Evidence ^a^

Traditional Name ^b^	Scientific Name	Family	Temperament ^b^	Traditional Indication	Modern Equivalent	Pharmacological Action	Safety/Toxicity
**Mezmaral-Rai**	*Alisma plantago* L.	Alismaceae	Hot and dry	Stomach ulcers, uterine pain, gallstones	Gastritis, dysmenorrhea, biliary colic	Antispasmodic, diuretic	Generally safe
**Koras**	*Allium* sp.	Alliaceae	Hot and dry	Prevents miscarriage, reduces uterine moisture	Threatened abortion, endometrial inflammation	Uterotonic, anti-inflammatory	Use with caution during pregnancy.
**Narjes**	*Narcissus tazetta* L.	Amaryllidaceae	Hot and dry to moderate	Aphrodisiac, wound healing, bladder pain, abortifacient	Libido enhancer, uterine analgesia	Neuroactive alkaloids, uterotonic	High toxicity - not recommended clinically
**Sumac**	*Rhus coriaria *L.	Anacardiaceae	Cold and dry	Diarrhea, uterine bleeding, discharge	Menorrhagia, leukorrhea	Astringent, anti-inflammatory	Generally safe
**Phostoge**	*Pistacia vera* L.	Anacardiaceae	Hot and dry	Prevents colds, uterine pain	Uterine cramping, dysmenorrhea	Analgesic, anti-inflammatory	Generally safe
**Mastaki**	*P. chia* DC.	Anacardiaceae	Hot and dry	Uterine pain, gonorrhea, cold uterus	Uterine atony, reproductive tract infection	Antispasmodic, antimicrobial	Generally safe
**Bakhor al-Akrad**	*Peucedanum officinale* L.	Apiaceae	Hot and dry	Diarrhea, flatulence, bladder pain	IBS, cystitis, endometritis	Carminative, anti-inflammatory	Generally safe
**Jawshir**	*Opopanax chironium* (L.) Koch.	Apiaceae	Hot and dry	Barren uterus, menstruation, constipation, abortion	Amenorrhea, uterine fibroids	Emmenagogue, antispasmodic	Use with caution in pregnancy.
**Duchess**	*Athamanta cretensis* L.	Apiaceae	Hot and dry	Uterine tonic, uterine enema, pregnancy support	Uterine cleansing, fertility support	Uterotonic, anti-inflammatory	Traditional use only; limited data
**Razyane**	*Hippomarathrum libanotis* Koch.	Apiaceae	Hot and dry	Leukorrhea, uterine disorders, anti-poison	Menstrual regulation, dysmenorrhea	Estrogenic, antispasmodic	Generally safe, avoid high doses in pregnancy
**Rai El Abel**	*Pastinaca sativa* L.	Apiaceae	Hot and dry	Emollient, anti-inflammatory, antidote for animal poison, relieves toothache and dyspnea; seeds used to treat leukorrhea	Leukorrhea, uterine inflammation, respiratory relief	Emollient, anti-inflammatory, detoxifying	Generally safe
**Sisalius**	*Seselitortuosum* L.	Apiaceae	Hot and dry	Stomach ulcers, uterine pain, gallstones	Gastritis, dysmenorrhea, biliary colic	Antispasmodic, diuretic	Generally safe
**Shabbat**	*Anethum graveolens* L.	Apiaceae	Hot and dry	Prevents miscarriage, reduces uterine moisture	Threatened abortion, endometrial inflammation	Uterotonic, anti-inflammatory	Use with caution during pregnancy.
**Korea**	*Carum carvi* L.	Apiaceae	Hot and dry	Aphrodisiac, wound healing, bladder pain, abortifacient	Libido enhancer, uterine analgesia	Neuroactive alkaloids, uterotonic	High toxicity - not recommended clinically
**Defli, Khazarhara**	*Nerium oleander* L. [high toxicity - not recommended for clinical use]	Apocynaceae	Hot and dry	Diarrhea, uterine bleeding, discharge	Menorrhagia, leukorrhea	Astringent, anti-inflammatory	Generally safe
**Jaidar**	*Ilex aquifolium* L.	Aquifoliaceae	Cold and dry	Prevents colds, uterine pain	Uterine cramping, dysmenorrhea	Analgesic, anti-inflammatory	Generally safe
**Asaron**	*Asarum europaeum* L. [high toxicity – not recommended for clinical use]	Aristolochiaceae	Hot and dry	Uterine pain, gonorrhea, cold uterus	Uterine atony, reproductive tract infection	Antispasmodic, antimicrobial	Generally safe
**Zarawand**	*Aristolochia longa* L.	Aristolochiaceae	Hot and dry	Diarrhea, flatulence, bladder pain	IBS, cystitis, endometritis	Carminative, anti-inflammatory	Generally safe
**Absinthin**	*Artemisia absinthium* L.	Asteraceae	Hot and dry	Barren uterus, menstruation, constipation, abortion	Amenorrhea, uterine fibroids	Emmenagogue, antispasmodic	Use with caution in pregnancy.
**Bahman**	*Centaurea behen* L.	Asteraceae	Hot and dry	Uterine tonic, uterine enema, pregnancy support	Uterine cleansing, fertility support	Uterotonic, anti-inflammatory	Traditional use only; limited data
**Daronaj**	*Doronicum roylei* DC. *D. grandiflorum* Lam.	Asteraceae	Hot and dry	Leukorrhea, uterine disorders, anti-poison	Menstrual regulation, dysmenorrhea	Estrogenic, antispasmodic	Generally safe, avoid high doses in pregnancy
**Qaisum**	*A. abrotonon* L.	Asteraceae	Hot and dry	Useful for chest pain, dyspnea, intestinal worms, diuresis, menstruation, and uterine disorders	Dysmenorrhea, uterine inflammation, parasite infections	Emmenagogue, antispasmodic, anthelmintic	Generally safe
**Gole davodi**	*Chrysanthemumindicum* L.	Asteraceae	Hot and dry	Stomach ulcers, uterine pain, gallstones	Gastritis, dysmenorrhea, biliary colic	Antispasmodic, diuretic	Generally safe
**Lahieh Altheis**	*Tragopogon lamottei* Rouy	Asteraceae	Moderate	Prevents miscarriage, reduces uterine moisture	Threatened abortion, endometrial inflammation	Uterotonic, anti-inflammatory	Use with caution during pregnancy.
**Mondi**	*Sphaeranthus africanus* L.	Asteraceae	Hot and humid	Aphrodisiac, wound healing, bladder pain, abortifacient	Libido enhancer, uterine analgesia	Neuroactive alkaloids, uterotonic	High toxicity - not recommended clinically
**Babonaj**	*Anthemis nobilis* L.	Asteraceae	Hot and dry	Diarrhea, uterine bleeding, discharge	Menorrhagia, leukorrhea	Astringent, anti-inflammatory	Generally safe
**Anbarbaris**	*Berberis vulgaris* L.	Berberidaceae	cold and dry	Prevents colds, uterine pain	Uterine cramping, dysmenorrhea	Analgesic, anti-inflammatory	Generally safe
**Abu Khalsa**	*Anchusa tinctoria* L.	Boraginaceae	Hot and dry	Uterine pain, gonorrhea, cold uterus	Uterine atony, reproductive tract infection	Antispasmodic, antimicrobial	Generally safe
**Khobbeh**	*Sisymbrium officinale* L.	Brassicaceae	Hot and humid	Diarrhea, flatulence, bladder pain	IBS, cystitis, endometritis	Carminative, anti-inflammatory	Generally safe
**Qonnabri**	*Lepidium draba* L.	Brassicaceae	Hot and dry	Barren uterus, menstruation, constipation, abortion	Amenorrhea, uterine fibroids	Emmenagogue, antispasmodic	Use with caution in pregnancy.
**Khardel**	*Brassica juncea* (L.) Czern.	Brassicaceae	Hot and dry	Uterine tonic, uterine enema, pregnancy support	Uterine cleansing, fertility support	Uterotonic, anti-inflammatory	Traditional use only; limited data
**Condor**	*Boswellia carteri* Birdw.	Burseraceae	Hot and dry	Leukorrhea, uterine disorders, anti-poison	Menstrual regulation, dysmenorrhea	Estrogenic, antispasmodic	Generally safe, avoid high doses in pregnancy
**Ghennab**	*Cannabis sativa* L. [moderate toxicity – use with caution.]	Cannabaceae	Hot and dry	Oil form treats earache, nerve pain, and endometrial inflammation; oral intake dries semen	Endometrial inflammation, neuropathic pain, reduced libido	Analgesic, anti-inflammatory, anti-secretory	Moderate toxicity – use with caution.
**Khaman**	*Sambucuse ebulus* L.	Caprifoliaceae	Cold and dry	Stomach ulcers, uterine pain, gallstones	Gastritis, dysmenorrhea, biliary colic	Antispasmodic, diuretic	Generally safe
**Satronion**	*Saponaria officinalis* L.	Caryophyllaceae	Hot and dry	Prevents miscarriage, reduces uterine moisture	Threatened abortion, endometrial inflammation	Uterotonic, anti-inflammatory	Use with caution during pregnancy.
**Turbot e hendi**	*Stictocardia tiliifolia* (Desr.) Hallier f.	Convolvulaceae	Hot and dry	Aphrodisiac, wound healing, bladder pain, abortifacient	Libido enhancer, uterine analgesia	Neuroactive alkaloids, uterotonic	High toxicity - not recommended clinically
**Kushous**	*Cuscuta epilinum* Mur.	Convolvulaceae	Hot and dry	Diarrhea, uterine bleeding, discharge	Menorrhagia, leukorrhea	Astringent, anti-inflammatory	Generally safe
**Ebron**	*Sempervivum arboreum* L.	Crassulaceae	Hot and dry	Prevents colds, uterine pain	Uterine cramping, dysmenorrhea	Analgesic, anti-inflammatory	Generally safe
**Hanzala, Abu Jahl watermelon**	*Cucumis colocynthis* L.	Cucurbitaceae	Hot and dry	Uterine pain, gonorrhea, cold uterus	Uterine atony, reproductive tract infection	Antispasmodic, antimicrobial	Generally safe
**Suad**	*Cyperus longus* L.	Cyperaceae	Hot and dry	Diarrhea, flatulence, bladder pain	IBS, cystitis, endometritis	Carminative, anti-inflammatory	Generally safe
**Ebons**	*Diospyros kaki* L.	Ebenaceae	Hot and dry	Barren uterus, menstruation, constipation, abortion	Amenorrhea, uterine fibroids	Emmenagogue, antispasmodic	Use with caution in pregnancy.
**Amsokh**	*Equisetum arvense* L.	Equisetaceae	Cold and dry	Uterine tonic, uterine enema, pregnancy support	Uterine cleansing, fertility support	Uterotonic, anti-inflammatory	Traditional use only; limited data
**Qutlub**	*Arbutus unedo* L.	Ericaceae	Cold and dry	Leukorrhea, uterine disorders, anti-poison	Menstrual regulation, dysmenorrhea	Estrogenic, antispasmodic	Generally safe, avoid high doses in pregnancy
**Afrabion**	*Euphorbia helioscopia* L.	Euphorbiaceae	Hot and dry	Removes phlegm, strengthens uterine tone, eliminates uterine odor	Uterine atony, abnormal vaginal discharge	Uterotonic, mucolytic, deodorizing	Use with caution (potentially toxic).
**Omghilan: Mughilan in Persian**	*Acacia nilotica* (L.) Willd.ex Delile	Fababceae	Cold and dry	Stomach ulcers, uterine pain, gallstones	Gastritis, dysmenorrhea, biliary colic	Antispasmodic, diuretic	Generally safe
**Bandag**	*Caesalpinia bonduc* Roxb.	Fabaceae	Hot and dry	Prevents miscarriage, reduces uterine moisture	Threatened abortion, endometrial inflammation	Uterotonic, anti-inflammatory	Use with caution during pregnancy.
**Holb**	*Trigonellafoenum-graecum *L.	Fabaceae	Hot and dry	Aphrodisiac, wound healing, bladder pain, abortifacient	Libido enhancer, uterine analgesia	Neuroactive alkaloids, uterotonic	High toxicity - not recommended clinically
**Hemmus**	*Cicer arietinum *L.	Fabaceae	Hot and dry	Diarrhea, uterine bleeding, discharge	Menorrhagia, leukorrhea	Astringent, anti-inflammatory	Generally safe
**Dar Shishaan**	*Calycotome spinosa *L.	Fabaceae	Hot and dry	Prevents colds, uterine pain	Uterine cramping, dysmenorrhea	Analgesic, anti-inflammatory	Generally safe
**Ballut,**	*Quercus ballota *Desf.	Fagaceae	Cold and dry	Uterine pain, gonorrhea, cold uterus	Uterine atony, reproductive tract infection	Antispasmodic, antimicrobial	Generally safe
**Irsa**	*Iris ensata* Thunb., *I. germanica* L.	Iridaceae	Hot and dry	Diarrhea, flatulence, bladder pain	IBS, cystitis, endometritis	Carminative, anti-inflammatory	Generally safe
**Susan**	*I. versicolor* L.	Iridaceae	Hot and dry	Barren uterus, menstruation, constipation, abortion	Amenorrhea, uterine fibroids	Emmenagogue, antispasmodic	Use with caution in pregnancy.
**Acl: Kulan**	*Juncus acutus* L.	Juncaceae	Cold and wet	Uterine tonic, uterine enema, pregnancy support	Uterine cleansing, fertility support	Uterotonic, anti-inflammatory	Traditional use only; limited data
**Juda**	*Teucrium polium* L. [moderate toxicity – use with caution.]	Lamiaceae	Hot and dry	Leukorrhea, uterine disorders, anti-poison	Menstrual regulation, dysmenorrhea	Estrogenic, antispasmodic	Generally safe, avoid high doses in pregnancy.
**Rasen**	*Calamintha incana* Boiss. & Held.	Lamiaceae	Hot and dry	Beneficial for heart, stomach, digestion, and bladder; acts as antidote and tonic; decoction induces menstruation	Digestive weakness, dysmenorrhea, fatigue	Emmenagogue, carminative, cardiotonic	Generally safe – avoid high doses.
**Sisnaber**	*Mentha piperita* L.	Lamiaceae	Hot and dry	Stomach ulcers, uterine pain, gallstones	Gastritis, dysmenorrhea, biliary colic	Antispasmodic, diuretic	Generally safe
**Ghastaron**	*Stachys officinalis* (L.) Trevis	Lamiaceae	Hot and dry	Prevents miscarriage, reduces uterine moisture	Threatened abortion, endometrial inflammation	Uterotonic, anti-inflammatory	Use with caution during pregnancy.
**Komaphytus**	*Ajuga chamaepitys* (L.) Schreb.	Lamiaceae	Hot and dry	Aphrodisiac, wound healing, bladder pain, abortifacient	Libido enhancer, uterine analgesia	Neuroactive alkaloids, uterotonic	High toxicity - not recommended clinically
**Marmahoz**	*Origanum maru* L.	Lamiaceae	Hot and dry	Diarrhea, uterine bleeding, discharge	Menorrhagia, leukorrhea	Astringent, anti-inflammatory	Generally safe
**Marmazad**	*S. lavandulifolia* Vahl.	Lamiaceae	Hot and dry	Prevents colds, uterine pain	Uterine cramping, dysmenorrhea	Analgesic, anti-inflammatory	Generally safe
**Kamazarios**	*T. chamaedrys* L.	Lamiaceae	Hot and dry	Uterine pain, gonorrhea, cold uterus	Uterine atony, reproductive tract infection	Antispasmodic, antimicrobial	Generally safe
**Saazaj**	*Cinnamomum citriodorum* Thwaites.	Lauraceae	Hot and dry	Diarrhea, flatulence, bladder pain	IBS, cystitis, endometritis	Carminative, anti-inflammatory	Generally safe
**Salikha**	*C. bejolghota* (Buch. -Ham.) Sweet	Lauraceae	Hot and dry	Barren uterus, menstruation, constipation, abortion	Amenorrhea, uterine fibroids	Emmenagogue, antispasmodic	Use with caution in pregnancy.
**Baheshtan**	*Laurus nobilis* L.	Lauraceae	Hot and dry	Uterine tonic, uterine enema, pregnancy support	Uterine cleansing, fertility support	Uterotonic, anti-inflammatory	Traditional use only; limited data
**Sonbole**	*Hyacinthus orientalis* L.	Liliaceae	Cold and dry	Leukorrhea, uterine disorders, anti-poison	Menstrual regulation, dysmenorrhea	Estrogenic, antispasmodic	Generally safe, avoid high doses in pregnancy.
**Kattan**	*Linum catharticum* L.	linaceae	cold and dry	Incense relieves nasal congestion and colds; Sitz bath and fumigation benefit uterus, wounds, and leukorrhea	Leukorrhea, uterine inflammation, sinusitis	Anti-inflammatory, mucolytic, wound healing	Generally safe
**Khubazi**	*Malva* sp.	Malvaceae	Cold and wet	Stomach ulcers, uterine pain, gallstones	Gastritis, dysmenorrhea, biliary colic	Antispasmodic, diuretic	Generally safe
**Khatmi**	*Althaea ficifolia* L.	Malvaceae	Cold and wet	Prevents miscarriage, reduces uterine moisture	Threatened abortion, endometrial inflammation	Uterotonic, anti-inflammatory	Use with caution during pregnancy.
**Ghatton**	*Gossypium herbaceum* L.	Malvaceae	Hot and dry	Aphrodisiac, wound healing, bladder pain, abortifacient	Libido enhancer, uterine analgesia	Neuroactive alkaloids, uterotonic	High toxicity - not recommended clinically
**Tin**	*Ficus carica* L.	Moraceae	Hot and humid	Diarrhea, uterine bleeding, discharge	Menorrhagia, leukorrhea	Astringent, anti-inflammatory	Generally safe
**Ace Bastani**	*Myrtus communis* L.	Myrtaceae	Cold	Prevents colds, uterine pain	Uterine cramping, dysmenorrhea	Analgesic, anti-inflammatory	Generally safe
**Gharnaphole**	*Syzygium aromaticum* (L.) Merr. & L. M. Perry	Myrtaceae	Hot and dry	Uterine pain, gonorrhea, cold uterus	Uterine atony, reproductive tract infection	Antispasmodic, antimicrobial	Generally safe
**Nilofer**	*Nymphaea alba* L.	Nymphaeaceae	Cold and dry	Diarrhea, flatulence, bladder pain	IBS, cystitis, endometritis	Carminative, anti-inflammatory	Generally safe
**Zeytoon**	*Olea europaea* L.	Oleaceae	Hot and dry	Barren uterus, menstruation, constipation, abortion	Amenorrhea, uterine fibroids	Emmenagogue, antispasmodic	Use with caution in pregnancy.
**Lasan al-Asafir**	*Fraxinus excelsior* L.	Oleaceae	Hot and dry	Uterine tonic, uterine enema, pregnancy support	Uterine cleansing, fertility support	Uterotonic, anti-inflammatory	Traditional use only; limited data
**Sanobar**	*Pinus eldarica* Medew.	Pinaceae	Hot and dry	Leukorrhea, uterine disorders, anti-poison	Menstrual regulation, dysmenorrhea	Estrogenic, antispasmodic	Generally safe, avoid high doses in pregnancy.
**Arze**	*Oryza sativa* L.	Poaceae	Moderate	Treats kidney and bladder diseases, uterine asphyxia, and supports sperm production	Renal and bladder disorders, male infertility	Nutritive, restorative, genitourinary tonic	Generally safe
**Hammaze**	*Rumex acetosa* L.	Polygonaceae	Cold and dry	Stomach ulcers, uterine pain, gallstones	Gastritis, dysmenorrhea, biliary colic	Antispasmodic, diuretic	Generally safe
**Gharighun**	*Polyporus officinalis* Fr.	Polyporaceae	Hot and dry	Prevents miscarriage, reduces uterine moisture	Threatened abortion, endometrial inflammation	Uterotonic, anti-inflammatory	Use with caution during pregnancy.
**Baqalah al-Hamqa**	*Portulaca oleracea* L.	Portulacaceae	Cold and wet	Aphrodisiac, wound healing, bladder pain, abortifacient	Libido enhancer, uterine analgesia	Neuroactive alkaloids, uterotonic	High toxicity - not recommended clinically
**Kharabq Asud**	*Helleborus niger* L.	Ranunculaceae	Hot and dry	Diarrhea, uterine bleeding, discharge	Menorrhagia, leukorrhea	Astringent, anti-inflammatory	Generally safe
**Zornabad**	*Delphinium semibarbatum* Bi.	Ranunculaceae	Hot and humid	Prevents colds, uterine pain	Uterine cramping, dysmenorrhea	Analgesic, anti-inflammatory	Generally safe
**Aligh**	*Rubus fructicosus* L.	Rosaceae	Cold and dry	Uterine pain, gonorrhea, cold uterus	Uterine atony, reproductive tract infection	Antispasmodic, antimicrobial	Generally safe
**Nasreen**	*Rosa canina* L.	Rosaceae	Hot and dry	Diarrhea, flatulence, bladder pain	IBS, cystitis, endometritis	Carminative, anti-inflammatory	Generally safe
**Sudab**	*Ruta graveolens* L. [moderate toxicity – use with caution.]	Rutaceae	Hot and dry	Barren uterus, menstruation, constipation, abortion	Amenorrhea, uterine fibroids	Emmenagogue, antispasmodic	Use with caution in pregnancy.
**Narenje**	*Citrus aurantium* L.	Rutaceae	Cold and dry	Uterine tonic, uterine enema, pregnancy support	Uterine cleansing, fertility support	Uterotonic, anti-inflammatory	Traditional use only; limited data
**Khelafe**	*Salix acmophylla* Boiss.	Salicaceae	Cold and dry	Leukorrhea, uterine disorders, anti-poison	Menstrual regulation, dysmenorrhea	Estrogenic, antispasmodic	Generally safe, avoid high doses in pregnancy.
**Arak Jabali**	*Salvadora persica* L.	Salvadoraceae	Hot and dry	Reduces endometrial inflammation and treats hemorrhoids	Endometritis, hemorrhoidal inflammation	Anti-inflammatory, mucosal astringent	Generally safe
**Banj or Bank**	*Hyoscyamus niger* L. [High Toxicty – Not recommended for clinical use]	Solanaceae	Cold and dry	Stomach ulcers, uterine pain, gallstones	Gastritis, dysmenorrhea, biliary colic	Antispasmodic, diuretic	Generally safe
**Yabrouh al-Sanam**	*Mandragora officinarum* L.	Solanaceae	Cold and dry	Prevents miscarriage, reduces uterine moisture	Threatened abortion, endometrial inflammation	Uterotonic, anti-inflammatory	Use with caution during pregnancy.
**Moghath**	*Glossostemon bruguieri* DC.	Sterculiaceae	Hot and dry	Aphrodisiac, wound healing, bladder pain, abortifacient	Libido enhancer, uterine analgesia	Neuroactive alkaloids, uterotonic	High toxicity - not recommended clinically
**Asl**	*Tamarix aphylla* (L.) Karst.	Tamaricaceae	Cold and dry	Diarrhea, uterine bleeding, discharge	Menorrhagia, leukorrhea	Astringent, anti-inflammatory	Generally safe
**Tarfa**	*T. gallica* L.	Tamaricaceae	Cold and dry	Prevents colds, uterine pain	Uterine cramping, dysmenorrhea	Analgesic, anti-inflammatory	Generally safe
**Oud**	*Aquilaria malaccessis* Lam.	Thymelaeaceae	Hot and dry	Uterine pain, gonorrhea, cold uterus	Uterine atony, reproductive tract infection	Antispasmodic, antimicrobial	Generally safe
**Nardin**	*Nardostachys jatamansi* D.C.	Valerianaceae	Hot and dry	Diarrhea, flatulence, bladder pain	IBS, cystitis, endometritis	Carminative, anti-inflammatory	Generally safe
**Aslaq**	*V. agnus-castus* L.	Verbenaceae	Hot and dry	Barren uterus, menstruation, constipation, abortion	Amenorrhea, uterine fibroids	Emmenagogue, antispasmodic	Use with caution in pregnancy.
**Rai al-Hamam**	*Verbena officinalis* L.	Verbenaceae	Hot and dry	Uterine tonic, uterine enema, pregnancy support	Uterine cleansing, fertility support	Uterotonic, anti-inflammatory	Traditional use only; limited data

^a^ Evidence strength reflects study quantity, clinical relevance, and consistency across pharmacological findings.

^b^ This information is sourced from the book Makhzan al-Adviya.

It is important to note that these temperament classifications reflect qualitative, humoral concepts rooted in PM, which describe the physiological state of the uterus in terms of four qualities: Hot, cold, wet, and dry. These categories serve as traditional diagnostic and therapeutic guides. While modern biomedical science does not directly measure these temperaments, approximate correlations can be drawn: For example, a “cold and wet” temperament may correspond to conditions involving hormonal insufficiency or increased inflammation, whereas “hot and dry” may reflect heightened uterine contractility or estrogenic dominance. These correlations are conceptual and should be interpreted cautiously, serving to bridge traditional medical paradigms with contemporary pathophysiological understanding.

**Figure 2.**
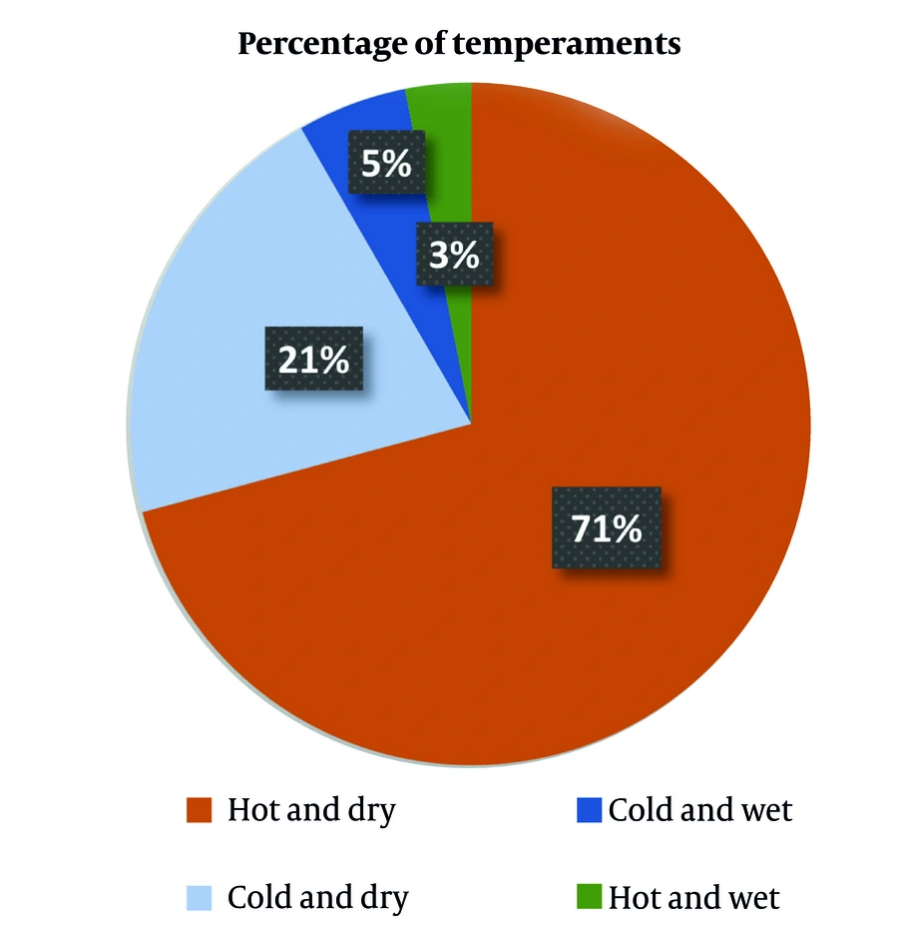
Distribution of traditional temperaments among uterine medicinal plants in Traditional Persian Medicine (TPM); colors represent major temperament types: Hot and dry (orange), cold and dry (light blue), cold and wet (dark blue), hot and wet (dark green).

According to Figure 2, approximately 71% of the plants traditionally prescribed for uterine diseases possess a hot and dry temperament, followed by 21% with hot and moist, 5% with cold and moist, and 3% with warm and moist temperaments. This indicates a traditional emphasis on using dry-tempered plants in the treatment of uterine disorders, possibly to counteract perceived moist imbalances in the female reproductive system.

**Figure 3.**
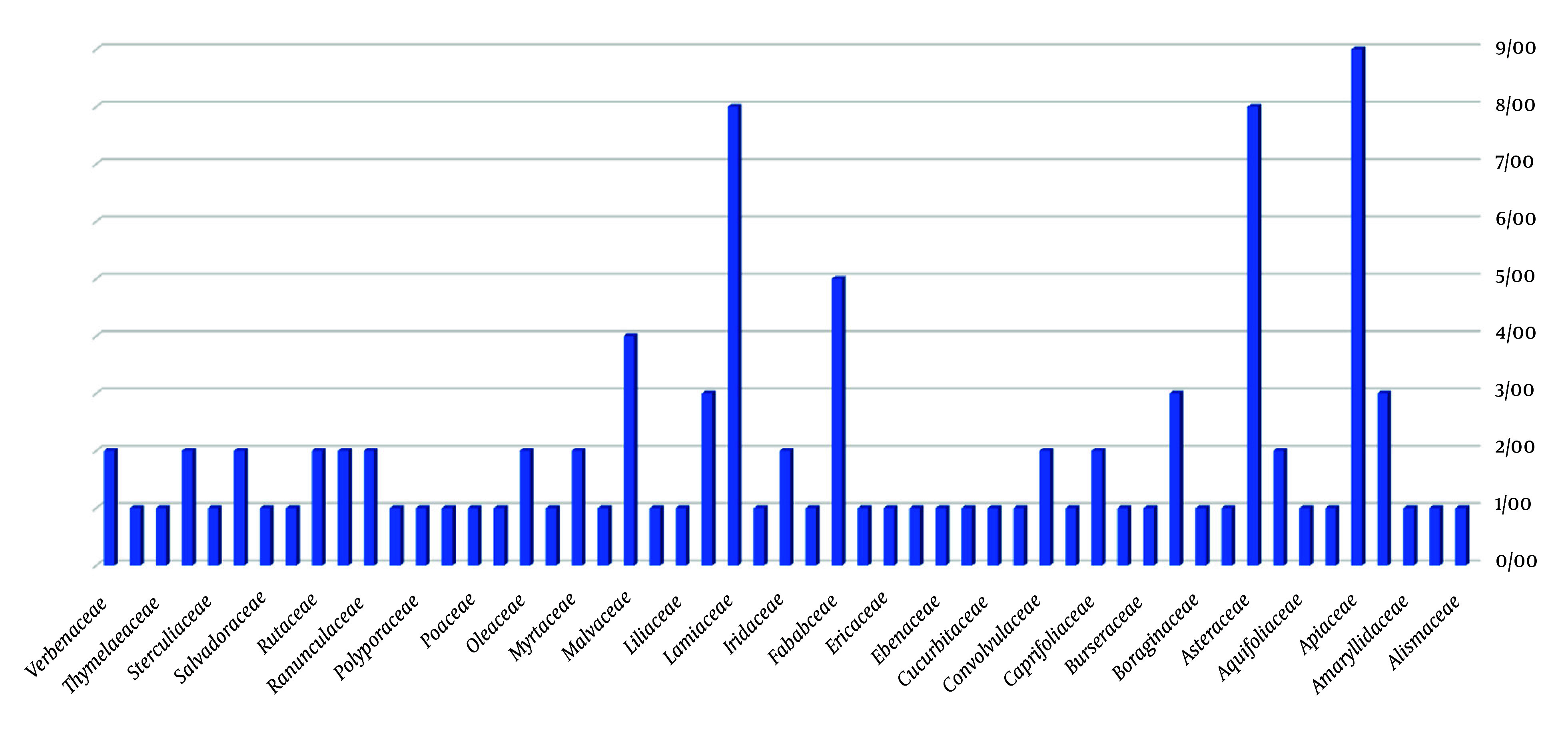
Percentage distribution of medicinal plant families used in the treatment of uterine diseases

In total, 39 plant families are represented in the treatment of uterine diseases. As shown in Figure 3, the most common families include Apiaceae, Asteraceae, and Lamiaceae, which are frequently used for conditions such as uterine pain, bloating, and moisture imbalance. For instance, *Saponaria officinalis* is used in uterine enemas to reduce moisture and hardness. Similarly, *Ilex aquifolium*, *Acacia nilotica*, and *Quercus ballota* are employed in formulations for uterine enemas. *Descurainia sophia* is traditionally used to support fetal development and strengthen the cervix during pregnancy.

The Lamiaceae family, characterized by predominantly hot and dry temperaments, includes several species applied to specific uterine conditions:

- Leukorrhea: *Cinnamomum bejolghota*, *Mentha piperita*.

- Amenorrhea: *Calamintha incana*.

- Uterine enema: *Teucrium polium*.

- Cervical stenosis: *Stachys officinalis*.

- Endometritis: *Ajuga chamaepitys*.

- Flatus vaginalis: *S. lavandulifolia*.

- Uterine flatulence: *T. chamaedrys*.

These examples illustrate the wide spectrum of medicinal plant applications for gynecological conditions in PM, with specific plants targeting distinct pathophysiological presentations.

**Figure 4.**
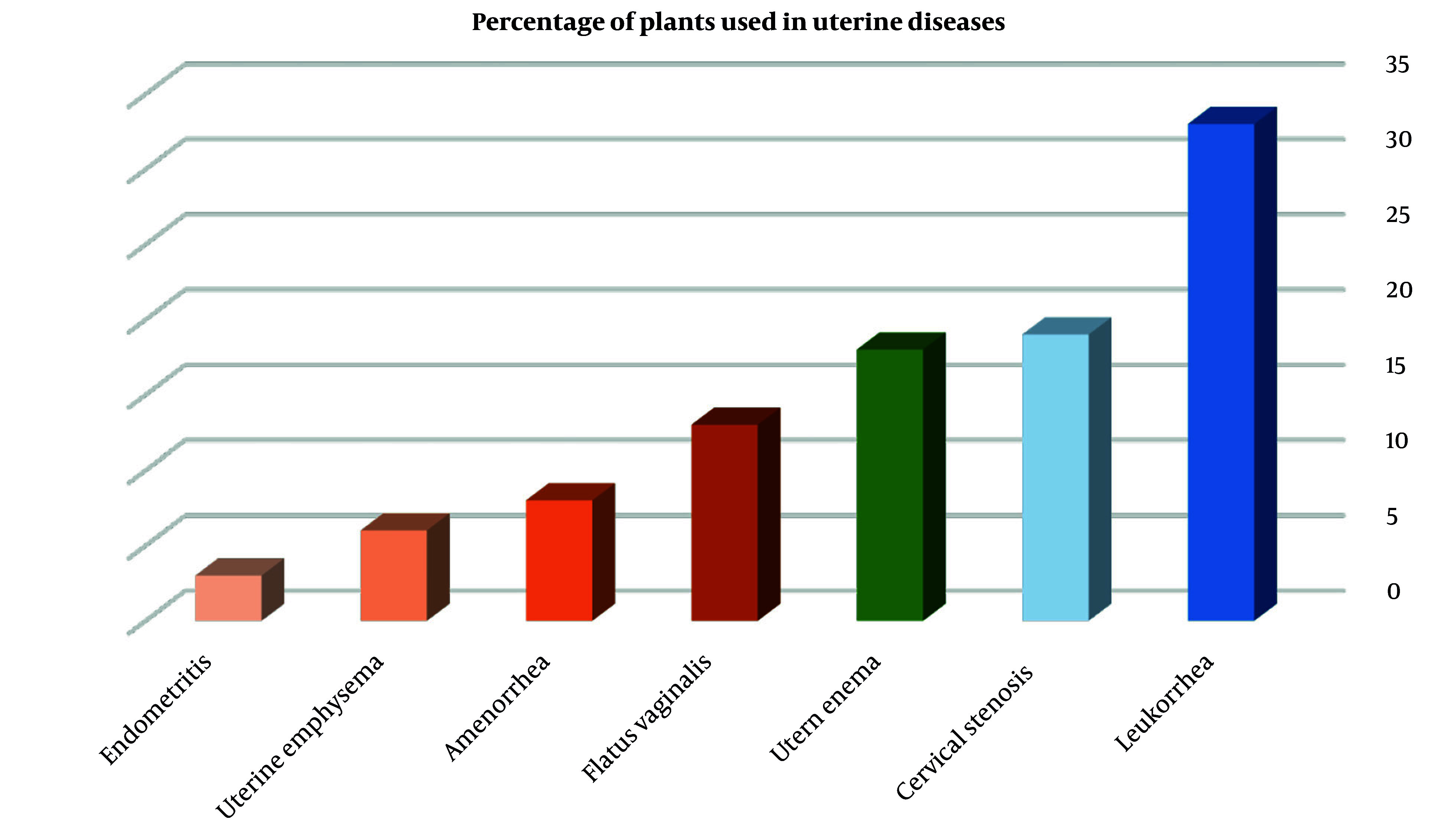
Percentage distribution of medicinal plants used in the treatment of various uterine disorders according to Traditional Persian Medicine (TPM). Columns are color-coded based on the predominant temperament of herbs used for each condition: Hot and dry (orange), cold and dry (blue), or cleansing/purifying agents (green).

Figure 4 summarizes the percentage distribution of traditional uses of the selected medicinal plants across various uterine conditions, including dysmenorrhea, infertility, amenorrhea, leukorrhea, and menstrual irregularities.

In line with reviewers’ recommendations to emphasize high-potential medicinal plants, a summary table entitled “Pharmacological Insights into Top Traditional Persian Medicinal Plants for Uterine Disorders” was prepared. Table 1 offers a concise synthesis of ten key medicinal plants traditionally used for gynecological conditions in PM. This table highlights their bioactive compounds, types of pharmacological evidence (in vitro, in vivo, or clinical), and documented therapeutic effects. The selection prioritizes plants with robust pharmacological backing, aiming to bridge traditional knowledge with contemporary scientific validation. This overview underscores both the therapeutic potential and the need for evidence-based evaluation to support the safe and effective use of these plants in women's reproductive health.

As shown in Table 3, several plants such as *Foeniculum vulgare* and *Cinnamomum* spp. are shared across traditional systems (“b” in Table 3), reflecting a convergence of empirical uses and therapeutic logic. Others, like *Nigella sativa* and *Ruta graveolens*, appear unique to TPM (“c” in Table 3), possibly due to regional availability or cultural emphasis.

**Table 3.** Comparative Use of Selected Medicinal Plants in Traditional Persian Medicine, Ayurveda, and Traditional Chinese Medicine for Treating Uterine Disorders ^a^

Plant Name (Scientific)	Use in TPM	Use in Ayurveda	Use in TCM	Form of Use/Notes
***Foeniculum vulgare*** ^**b**^	Emmenagogue, warms uterus, reduces uterine bloating	Used for dysmenorrhea, infertility, promotes lactation	Warms interior, regulates Qi, promotes menstrual flow	Seed decoction or powder; used widely in all systems
***Nigella sativa*** ^**c**^	Warms and strengthens uterus, treats hypoestrogenism	Fertility enhancer, uterine tonic	Less common; used as general tonic	Seed oil or roasted seed; prominent in TPM and Ayurveda
***Crocus sativus*** ^**d**^	Relieves dysmenorrhea, improves mood, regulates menses	Used for painful periods, PMS, mood balance	Activates blood, calms Shen (spirit), anti-depressant	Stigma infusion or powder; shared across all three traditions
***V** **.** ** agnus-** **castus*** ^**b**^	Hormonal balance, treats amenorrhea and PMS	Regulates menstruation, galactagogue	Rarely used	Standardized extract or dried fruit; common in TPM and Ayurveda
***Teucrium ** **polium*** ^**b**^	Tonifies uterus, reduces excess moisture/discharge	Limited mention	Not traditionally used	Aerial parts decoction; primarily TPM-specific
***Ruta graveolens*** ^**b**^	Emmenagogue, resolves uterine stagnation	Used in low doses for delayed menses, abortifacient effect	Considered toxic and rarely used	Used with caution due to toxicity; internal use limited in all systems
***Ajuga ** **chamaepitys*** ^**b**^	Uterine cleanser, inflammation	Not commonly used	Not used	Infusion or decoction; primarily oral use
***T. ** **polium*** ^**b**^	Uterine tonic, emmenagogue	Bitter tonic, general use	Reported hepatotoxicity	Oral infusion; traditional caution in liver disease

Abbreviations: TPM, Traditional Persian Medicine; TCM, Traditional Chinese Medicine.

^a^ The table highlights therapeutic indications and preparations of six key plants across three major traditional medical systems. Differences in usage, safety, and formulation reflect both shared and divergent ethnomedical principles.

^b^ Shared use across TPM, TCM, and Ayurveda.

^c^ Traditional use reported only in TPM.

^d^ Reported in both TPM and Ayurveda, not in TCM.

## 4. Discussion

**Figure 5.**
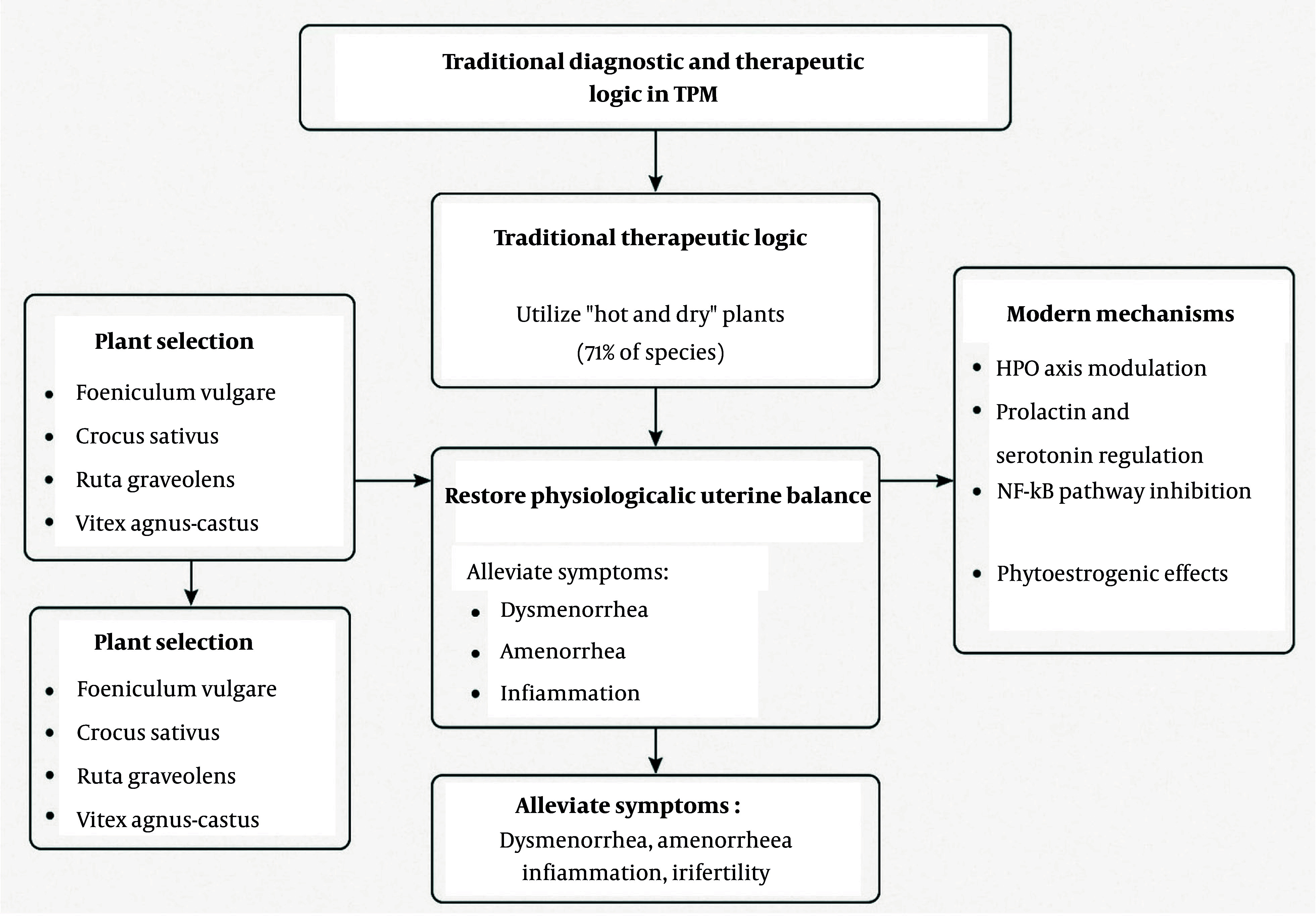
Conceptual illustration of the relationship between traditional Persian temperament theory and modern physiological interpretations of uterine disorders

This review comprehensively investigates medicinal plants from PM used for uterine disorders, emphasizing traditional uses, temperament classifications, and scientific validations. The PM, rooted in humoral theory, maintains health through the balance of four qualities: Hot, cold, wet, and dry. Uterine disorders are often attributed to an excess of cold and wet humors, commonly referred to as “cold uterine temperament”. Treatments thus focus on substances with warming and drying effects. This theoretical framework is reflected in the current findings, where 71% of the identified medicinal plants exhibit a “hot and dry” temperament, supporting the intended therapeutic rationale of counteracting cold-wet imbalances (Figure 5). 

Among the 97 documented species, plants such as *F. vulgare*, *N. sativa*, *Cuminum cyminum*, *Crocus sativus*, and *R. graveolens* were frequently cited for gynecological conditions (29, 31-33). Pharmacological findings further support the traditional use of these species. For instance, *F. vulgare*, rich in trans-anethole and fenchone, exhibits estrogenic and antispasmodic effects that may contribute to menstrual regulation (34). Key medicinal plants such as *N. sativa*, *F. vulgare*, *C. sativus*, and *Vitex agnus-castus* exert their effects through diverse molecular pathways. Thymoquinone from *N. sativa* modulates prostaglandin synthesis and calcium signaling while suppressing inflammation via NF-κB inhibition. *Foeniculum vulgare* may influence the hypothalamic-pituitary-ovarian (HPO) axis, supporting hormonal regulation. *Crocus sativus* components like crocin and safranal affect serotonin and dopamine systems, contributing to mood and endocrine balance. *Vitex agnus-castus* acts on dopamine D2 receptors, lowering prolactin levels and alleviating luteal phase dysfunctions (35, 36).

Despite these promising profiles, several safety concerns arise. Some traditionally cited species, such as *Aristolochia* spp. and *Nerium oleander*, are known to be hepatotoxic or nephrotoxic and thus warrant cautious interpretation (37, 38). For instance, *Aristolochia* spp. contain aristolochic acids — nephrotoxic and carcinogenic compounds — that have led to regulatory bans in multiple countries. Likewise, *N. oleander* contains cardiac glycosides that are potentially lethal even in small doses. *Cannabis sativa*, although showing therapeutic potential, poses challenges due to psychoactive effects and legal constraints in many jurisdictions (39). Therefore, the inclusion of such species in PM demands critical toxicological evaluation and should be avoided in clinical practice without comprehensive safety data.

When compared to modern gynecological therapies — such as NSAIDs, hormonal treatments, and surgical options — PM offers a holistic and individualized approach, emphasizing systemic balance and long-term well-being. Some PM remedies, such as *F. vulgare* and *C. sativus*, have demonstrated effects that are comparable or complementary to modern pharmaceuticals in managing conditions like dysmenorrhea and depression. However, variability in plant preparation methods, lack of dosage standardization, and safety uncertainties currently limit their integration into evidence-based care. A synergistic model combining PM insights with scientific rigor could pave the way for more holistic and accessible approaches in reproductive health.

A focused analysis of the top ten medicinal plants with pharmacological evidence (Table 1) revealed that five species — *A. absinthium*, *R. graveolens*, *V. agnus-castus*, *A. chamaepitys*, and *T. polium* — are consistently cited in classical TPM sources and pharmacological studies as being primarily used for uterine health. These plants are traditionally employed for specific indications such as endometrial inflammation, menstrual irregularities, endometritis, and uterine tonicity. The inclusion of a new column titled “Primary Uterine Use” in Table 1 helps differentiate these targeted herbs from those with more general applications. This classification enhances the precision of our ethnopharmacological assessment and provides a rational basis for selecting lead candidates for clinical research focused on women’s reproductive health.

In addition to widely studied plants like *N. sativa* and *V. agnus-castus*, certain lesser-known species also emerged as noteworthy. For instance, *A. chamaepitys* and *T. polium* are traditionally used in TPM for treating uterine inflammation and discharge, yet they remain underrepresented in modern pharmacological research. Given their specific uterine indications and ethnomedical significance, these species warrant priority in future bioactivity screening, safety assessments, and clinical validation.

A phytochemical overview of the reviewed plants reveals that flavonoids, terpenoids, alkaloids, and phenolic acids are the dominant compound classes associated with uterine pharmacological actions. These compounds have been linked to anti-inflammatory, spasmolytic, and hormone-regulating properties. For instance, *F. vulgare* contains anethole, which mimics estrogenic activity; *N. sativa* is rich in thymoquinone, a potent antioxidant and uterine relaxant; and *V. agnus-castus* produces iridoids and diterpenes that affect pituitary hormone regulation. This phytochemical landscape provides a biochemical basis for the empirical efficacy of TPM remedies in women's reproductive health.

Table 3 presents a comparative analysis of selected medicinal plants used for uterine disorders in TPM, Ayurveda, and TCM. Plants such as *F. vulgare* and *C. sativus* demonstrate cross-cultural consensus regarding their uterine applications, while species like *T. polium* remain more specific to TPM. Such differences stem from diagnostic paradigms — humoral imbalance in TPM, dosha theory in Ayurveda, and Qi-based frameworks in TCM. Recognizing these contrasts enhances the interpretive bridge between traditional knowledge systems and supports culturally sensitive pharmacological research.

Some traditional uterine remedies mentioned in classical sources include potent abortifacients or toxic agents such as *R. graveolens*, *Peganum harmala*, and *Aristolochia* spp. The modern clinical use of such herbs raises ethical and legal concerns. For example, *C. sativa* is prohibited in certain countries for reproductive use, and *Aristolochia* species are banned by the EMA and WHO due to nephrotoxicity and carcinogenicity. Furthermore, the cultural context surrounding the use of abortifacient herbs necessitates heightened sensitivity and ethical consideration. Given the potential for serious adverse effects, these plants should only be employed with comprehensive patient education, clear labeling, informed consent, and under professional supervision. This is particularly critical for women of reproductive age, where uninformed or inappropriate use could lead to harmful outcomes. Therefore, herbs with emmenagogue or abortifacient actions should be approached with extreme caution. Future ethnopharmacological research should incorporate toxicological screening and respect international regulatory frameworks to ensure the safe and responsible application of these medicinal plants.

To facilitate clinical translation and regulatory acceptance, there is a pressing need to develop standardized botanical formulations for high-priority medicinal plants such as *F. vulgare*, *N. sativa*, and *V. agnus-castus*. Traditional preparations often vary in method, dosage, and concentration of active constituents, leading to inconsistent therapeutic outcomes. Investment in producing standardized extracts — defined by quantified levels of bioactive markers and validated manufacturing protocols — can enhance reproducibility in clinical trials and pave the way for their inclusion in pharmacopeias and evidence-based guidelines for reproductive health.

Despite growing interest in integrating traditional Persian medicinal plants into modern gynecological care, a major limitation remains the lack of pharmacokinetic (PK) and dose-response (PD) data for many of the reviewed species. Without such data, it is difficult to determine optimal dosing, bioavailability, metabolism, and toxicity thresholds. This scientific gap not only hinders standardization but also poses challenges to regulatory approval and clinical translation. Future research should prioritize PK/PD profiling using modern analytical tools such as LC-MS/MS, alongside in vivo studies, to ensure accurate dosing, improved safety margins, and better therapeutic predictability of these traditional remedies.

In future clinical investigations, particular emphasis should be placed on safety monitoring, especially for plants with known or suspected toxicity. Standardized safety protocols should include the assessment of hepatic, renal, and reproductive toxicity markers throughout the trial period. For example, herbs such as *T. polium* and *R. graveolens* — associated with hepatotoxicity or abortifacient effects — should undergo comprehensive toxicological screening before clinical application. Such safety-focused approaches are crucial to minimize adverse effects and support the responsible integration of traditional herbal remedies into mainstream reproductive healthcare.

To improve the precision and objectivity of future studies, it is recommended to incorporate biomarker-guided approaches. Measurement of hormonal markers such as estrogen, follicle-stimulating hormone (FSH), and luteinizing hormone (LH), as well as inflammatory cytokines, can provide quantifiable endpoints to evaluate the efficacy of herbal interventions. Such biomarkers can help clarify mechanisms of action, optimize dosing, and identify patient subgroups that are most likely to benefit from specific medicinal plants.

Future research should adopt a gender-inclusive and age-specific approach when evaluating medicinal plants for uterine disorders. Considering factors such as menopausal status, menstrual cycle phases, and hormonal fluctuations is critical to accurately assess efficacy and safety. Tailoring study designs to these variables can improve the clinical relevance and personalized applicability of traditional herbal therapies (40-42).

### 4.1. Conclusions

This review underscores the therapeutic potential of PM in managing uterine disorders, particularly through the use of plants with a “hot and dry” temperament to counteract cold-moist imbalances traditionally associated with gynecological conditions. While many PM herbs exhibit multi-systemic effects, identifying species with predominant uterine applications — such as *V. agnus-castus*, *R. graveolens*, and *T. polium* — provides a focused direction for drug discovery and integrative health strategies. These herbs represent high-priority candidates for pharmacological validation. Future studies should emphasize these species through standardized phytochemical profiling, toxicological evaluation, and rigorously designed clinical trials to ensure their safety and efficacy in women’s reproductive health.

To facilitate clinical translation and regulatory acceptance, there is a pressing need to develop standardized botanical formulations for high-priority medicinal plants such as *F. vulgare*, *N. sativa*, and *V. agnus-castus*. Traditional preparations often vary in method, dosage, and concentration of active constituents, leading to inconsistent therapeutic outcomes. Investment in producing standardized extracts — defined by quantified levels of bioactive markers and validated manufacturing protocols — can enhance reproducibility in clinical trials and pave the way for their inclusion in pharmacopeias and evidence-based guidelines for reproductive health.

Future research should focus not only on validating traditional uses but also on designing targeted studies to evaluate efficacy and safety. For example, randomized double-blind clinical trials on *F. vulgare* for dysmenorrhea or *V. agnus-castus* for PMS could confirm historical claims. Moreover, integrating pharmacogenomic tools or biomarker-based stratification may help identify subgroups of women who are most likely to benefit from plant-based therapies. Such approaches would enhance the precision, personalization, and translational potential of herbal medicine in gynecology.

## References

[1] Fisher C, Adams J, Hickman L, Sibbritt D (2016). The use of complementary and alternative medicine by 7427 Australian women with cyclic perimenstrual pain and discomfort: a cross-sectional study.. BMC Complement Altern Med..

[2] Arab F, Afzalaghayi M, Vakilzade AK, Ghorbanzade A (2015). Evaluation of the reasons behind the use of traditional medicine from the perspective of Mashhad medical students.. Avicenna J Phytomed..

[3] Ekor M (2014). The growing use of herbal medicines: issues relating to adverse reactions and challenges in monitoring safety.. Front Pharmacol..

[4] Sahoo KC, Tamhankar AJ, Johansson E, Stalsby Lundborg C (2014). Community perceptions of infectious diseases, antibiotic use and antibiotic resistance in context of environmental changes: a study in Odisha, India.. Health Expect..

[5] Mohagheghzadeh A, Zargaran A, Daneshamuz S (2011). Cosmetic sciences from ancient Persia.. Pharm Hist (Lond)..

[6] Zargaran A, Zarshenas MM, Mehdizadeh A, Mohagheghzadeh A (2013). Management of tremor in medieval Persia.. J Hist Neurosci..

[7] Moeini R, Gorji N, Ghods R, Mozaffarpur SA (2017). Quantitative and qualitative assessment of Persian medicine articles indexed in PubMed by the end of 2015.. J Babol Univ Med Sci..

[8] Avicenna H (2005). Al-qanoon fi al-Tibb (the canon of medicine).. Dare Ehia Attorath Al Arabi. Beirut..

[9] Mashhadi M, Saeidi A, Tansaz M, Bioos S, Tabarrai M, Darvish-Mofrad-Kashani Z (2023). Evaluating the Indices of Diagnosing Uterine Temperament in Persian Medicine: A Review Study.. Crescent J Med Biol Sci..

[10] Rabizadeh F, Mirian MS, Doosti R, Kiani-Anbouhi R, Eftekhari E (2022). Phytochemical Classification of Medicinal Plants Used in the Treatment of Kidney Disease Based on Traditional Persian Medicine.. Evid Based Complement Alternat Med..

[11] Tabish SA (2008). Complementary and Alternative Healthcare: Is it Evidence-based?. Int J Health Sci (Qassim)..

[12] Rabizadeh F, Mirian MS (2024). The Classification of Medicinal Plants used in Traditional Persian Medicine for the Treatment of Liver Disease based on Phytochemical Properties.. J Med Plants By-products..

[13] Satyavati GV, Raina MK, Sharma M (1987). Medicinal plants of India..

[14] Katewa SS (2009). Indigenous People and Forests: Perspectives of an Ethnobotanical Study from Rajasthan (India).. Herbal Drugs: Ethnomedicine to Modern Medicine..

[15] Balamurugan S, Vijayakumar S, Prabhu S, Morvin Yabesh JE (2018). Traditional plants used for the treatment of gynaecological disorders in Vedaranyam taluk, South India - An ethnomedicinal survey.. J Tradit Complement Med..

[16] Aghili Khorasani M (2009). [Makhzan-ol-Adviyah [storehouse of medicaments]..

[17] Ibn Jazlah Y, Taghvim A (2023). [Traditional and Complementary Medicine]..

[18] Ghahraman A, Okhovat AR (2002). [Matching names of ancient herbs, with scientific names]..

[19] Ghahraman A, Okhovat AR (2002). [Comparative description of ancient herbs]..

[20] Shidfar F, Ebrahimi SS, Hosseini S, Heydari I, Shidfar S, Hajhassani G (2012). The effects of Berberis vulgaris fruit extract on serum lipoproteins, apoB, apoA-I, homocysteine, glycemic control and total antioxidant capacity in type 2 diabetic patients.. Iran J Pharmaceut Res..

[21] Esmaeili A, Farahpour MR, Oryan A (2012). [Effectiveness of Artemisia absinthium ointment on wound healing in rats].. Veterinary Res Forum..

[22] Rahimi R, Shams-Ardekani MR, Abdollahi M (2010). [A review of the efficacy of traditional Iranian medicine for uterine disorders and potential mechanisms].. J Ethnopharmacol..

[23] van Die MD, Burger HG, Teede HJ, Bone KM (2013). Vitex agnus-castus extracts for female reproductive disorders: a systematic review of clinical trials.. Planta Med..

[24] Goyal H, Singla U, Gupta U, May E (2017). Role of cannabis in digestive disorders.. European J Gastroenterol Hepatol..

[25] Ranasinghe P, Pigera S, Premakumara GA, Galappaththy P, Constantine GR, Katulanda P (2013). Medicinal properties of 'true' cinnamon (Cinnamomum zeylanicum): a systematic review.. BMC Complement Altern Med..

[26] Sharififar F, Dehghn-Nudeh G, Mirtajaldini. M (2009). Major flavonoids with antioxidant activity from Ajuga chamaecistus ssp. tomentella.. Iran J Pharmaceut Res..

[27] Esmaeili MA, Sonboli A (2010). Antioxidant, free radical scavenging activities of Salvia brachyantha and its protective effect against oxidative cardiac cell injury.. Food Chem Toxicol..

[28] Sayyah M, Hadidi N, Kamalinejad M (2004). Analgesic and anti-inflammatory activity of Lactuca sativa seed extract in rats.. J Ethnopharmacol..

[29] Ahmad A, Husain A, Mujeeb M, Khan SA, Najmi AK, Siddique NA (2013). A review on therapeutic potential of Nigella sativa: A miracle herb.. Asian Pac J Trop Biomed..

[30] Aziz MA, Khan AH, Ullah H, Adnan M, Hashem A, Abd_Allah EF (2018). Traditional phytomedicines for gynecological problems used by tribal communities of Mohmand Agency near the Pak-Afghan border area.. Revista Brasileira de Farmacognosia..

[31] Hasheminasab FS, Azimi M, Raeiszadeh M (2024). Therapeutic effects of saffron (Crocus sativus L) on female reproductive system disorders: A systematic review.. Phytother Res..

[32] Badgujar SB, Patel VV, Bandivdekar AH (2014). Foeniculum vulgare Mill: a review of its botany, phytochemistry, pharmacology, contemporary application, and toxicology.. Biomed Res Int..

[33] Dilip J, Jasmine G (2017). Pharmacological appraisal of cuminum cyminum l. In dysmenorrhoea: An ayurvedic approach in consideration of current evidences.. Int J Ayurveda Pharma Res..

[34] Rather MA, Dar BA, Sofi SN, Bhat BA, Qurishi MA (2016). Foeniculum vulgare: A comprehensive review of its traditional use, phytochemistry, pharmacology, and safety.. Arabian J Chem..

[35] Samadipour E, Rakhshani MH, Kooshki A, Amin B (2020). Local Usage of Nigella sativa Oil as an Innovative Method to Attenuate Primary Dysmenorrhea: A Randomized Double-blind Clinical Trial.. Oman Med J..

[36] Haddad B, Bidgolia SA, Qomic M, Asgarpanaha J (2018). Organ Toxicity and Estrogen Like Effects of Cuminum Cyminum.L Seed Essential Oil: A Hormonal, Histopathological and Immunohistochemical Study in Female Mice.. J Pharm Sci..

[37] Han J, Xian Z, Zhang Y, Liu J, Liang A (2019). Systematic Overview of Aristolochic Acids: Nephrotoxicity, Carcinogenicity, and Underlying Mechanisms.. Front Pharmacol..

[38] Farkhondeh T, Kianmehr M, Kazemi T, Samarghandian S, Khazdair MR (2020). Toxicity effects of Nerium oleander, basic and clinical evidence: A comprehensive review.. Hum Exp Toxicol..

[39] Whiting PF, Wolff RF, Deshpande S, Di Nisio M, Duffy S, Hernandez AV (2015). Cannabinoids for Medical Use: A Systematic Review and Meta-analysis.. JAMA..

[40] Sultana A, Rahman K (2022). Evaluation of general body temperament and uterine dystemperament in amenorrhoea: a cross-sectional analytical study.. J Complement Integr Med..

[41] Iranzadasl M, Bozorgi M, Pasalar M (2024). Contributions of Traditional Persian Medicine Lifestyle Principles in Primary Health Care: An Evidence-Based Review.. Shiraz E-Med J..

[42] Abdipour Mehrian SR, Uddin S, Ghahramani Z, Moshfeghinia R, Shahabi S, Haghdoost A (2024). Evidence-based practice in traditional persian medicine (TPM): a stakeholder and social network analysis.. BMC Complement Med Ther..

